# Effects of Anethole on Renal Function of Swiss Mice

**DOI:** 10.3390/ph18040541

**Published:** 2025-04-08

**Authors:** Romário Pinheiro-Lustosa, Neide Maria Silva Gondim-Pereira, Sarah Aparecida dos Santos Alves, Christina Maeda Takiya, Kerly Shamyra da Silva-Alves, Ana Acacia Sá Pinheiro, Andrelina Noronha Coelho-de-Souza, Maria Diana Moreira-Gomes, Celso Caruso-Neves, José Henrique Leal-Cardoso

**Affiliations:** 1 Superior Institute of Biomedical Sciences, Universidade Estadual do Ceará, Fortaleza 60714-903, Brazil; romario-lustosa@hotmail.com (R.P.-L.); neide.gondim@aluno.uece.br (N.M.S.G.-P.); kerly.shamyra@uece.br (K.S.d.S.-A.); andrelinanoronha@gmail.com (A.N.C.-d.-S.); dianammgr@hotmail.com (M.D.M.-G.); 2 Carlos Chagas Filho Institute of Biophysics, Universidade Federal do Rio de Janeiro, Rio de Janeiro 21941-617, Brazil; ssantos@biof.ufrj.br (S.A.d.S.A.); takiyacm@biof.ufrj.br (C.M.T.); acacia@biof.ufrj.br (A.A.S.P.); caruso@biof.ufrj.br (C.C.-N.)

**Keywords:** anethole, proximal tubule, tubulointerstitial injury, proteinuria, kidney, toxicity

## Abstract

**Background/Objectives:** Anethole, a terpenoid with several pharmacologic effects, is the major constituent of the essential oil of *Croton zehntneri* (EOCz), Pax & K. Hoffm, Euphorbiaceae. Due to the mild renal toxicity associated with high doses of EOCz, its potential therapeutic effects on several diseases, and the fact that its chemical composition consists of 80% anethole, the renal effects of anethole in mice were investigated. **Methods:** Mice were randomly divided into eight groups, dosed daily as follows: Group 1—CTRL (control; vehicle only); Groups 2—A100, 3—A125_2x_, and 4—A250 (dosed with 100, 125 twice daily, and 250 mg/kg, *per os* anethole); Group 5—_SUB_AKI (i.p. albumin to induce hyperproteinemia and proteinuria; subclinical acute kidney injury); and Groups 6—_SUB_AKI+A100, 7—_SUB_AKI+A125_2x_, and 8—_SUB_AKI+A250 (*per os* anethole + i.p. albumin). **Results:** The A125_2x_ and A250 groups significantly increased urinary proteinuria and interstitial inflammation (*p* < 0.001, for these groups). _SUB_AKI+A100, _SUB_AKI+A125_2x_, and _SUB_AKI+A250 showed a neither protective nor additive effect in the proteinuria induced by anethole and by administered albumin. The anethole-induced proteinuria was spontaneously reversible in approximately 4 weeks. In vitro experiments showed that anethole (300 µg/mL) inhibits albumin uptake from the culture medium by tubular cells. **Conclusions:** Anethole at high doses bears renal acute toxicity that, although mild and spontaneously fully reversible, must be taken into consideration in a cost–benefit analysis.

## 1. Introduction

Anethole (1-methoxy-4-(1-propenyl)-benzene) is a terpenoid that is present in several aromatic plants and has been receiving increasing attention in scientific pharmacological research [1,2,3,4]. Numerous studies have reported that anethole possesses anti-inflammatory effects in different acute [5,6,7] and chronic [8,9] inflammation models. Anethole also possesses antioxidant [10], anti-apoptotic [11], anti-metastatic [12], gastroprotective [13], antihypernociceptive [14], antihypertensive [15], and cicatricial activities [3]. Anethole protects the morphology and function of the kidney and liver exposed to hepatic and renal ischemia–reperfusion injury [11,16]. The essential oil of *Croton zehnteneri* Pax & K. Hoffm (EOCz), Euphorbiaceae, popularly named Canela de Cunhã, or Canelinha, or Canela de Cheiro in the Brazilian Northeast, containing approximately 80% anethole, exhibits significant pharmacological effects similar to those of anethole. Thus, EOCz has well-described pharmacological and biological activities, such as antinociceptive [17], antispasmodic [18], and larvicidal (against *Aedes aegypti*) activities [19], as well as being able to prevent diabetes mellitus-induced neuropathy [20,21].

Anethole is used in the food and pharmaceutical industries, and the United States Food and Drug Administration (FDA-US) has issued its safety certification [22,23]. Several experimental studies have shown that anethole has no toxicity at low doses [5,24]. It is considered non-genotoxic and non-carcinogenic and, therefore, quite safe [13,25]. Indeed, a sub-chronic *per os* treatment with EOCz and anethole in rats, lasting approximately 10 weeks, triggers renal toxicity at doses equal to or greater than 250 mg/kg. This toxicity is indicated by an increase in kidney weight relative to body weight, without any alteration in blood biochemical parameters [26].

However, recently, our group disclosed that EOCz, at 250 mg/kg, has mild toxic effects on renal function, leading to tubule-interstitial injury and proteinuria [27], diagnosed solely by proteinuria. This EOCz renal effect was very similar to that induced by hyperproteinemia in the acute kidney injury (AKI) model. Proteinuria is a pathognomonic parameter for the diagnosis of tubular injury (TI) at the initial stages [28,29]. Thus, the use of TI markers has become an interesting strategy to identify the initial stage of AKI, since an incomplete repair of TI during the course of AKI could lead to the development of chronic kidney disease (CKD) [28,29,30]. In this study, the subclinical AKI (_SUB_AKI) animal model was chosen to verify the interaction between anethole and hyperproteinemia-induced _SUB_AKI derangements, because it mimics tubular injury without glomerular changes [31,32,33,34].

Although the anti-inflammatory and potentially therapeutic effects of anethole are well documented, EOCz at moderately high doses (250 mg/kg) has demonstrated a mild renal toxicity like that characterized by hyperproteinemia, similar to that observed in the AKI model [27]. Since the effects of EOCz are not equal to those of anethole [18], and at the same doses anethole has demonstrated important anti-inflammatory effects and great potentiality for the treatment of diabetic neuropathy and other ailments, anethole was investigated. Given that EOCz, rich in anethole, has demonstrated therapeutic potential against several diseases, including diabetic neuropathy [20,21], and prolonged anethole use has been associated with changes in renal weight relative to body mass [18], it is essential to clarify its potential renal toxicity and underlying mechanisms—especially since these remain unexplored for EOCz. Therefore, hypothesizing that the renal toxicity of anethole is similar to those of EOCz, we investigated whether anethole (1) at the same dose (250 mg/kg) induces effects similar to those of the EOCz, the type of renal toxicity, and the mechanism of action; (2) at a smaller dose (100 mg/kg), still within the range of documented anti-inflammatory potency (1.0–30 mg/kg), induces any renal toxicity; and (3) whether this effect is spontaneously reversible.

## 2. Results

### 2.1. Effects of Anethole Treatment on Glomerular Renal Function

To determine the effects of treatment with high (250 mg/kg) and medium (100 mg/kg) doses on kidney function and morphology with a special interest in elucidating possible kidney toxicity, Swiss mice in a healthy condition or with tubular injury (AKI model) were treated. Eight groups were generated as described in the Materials and Methods Section (Section 4.2).

Body mass, absolute and relative kidney weight, food and water intake, and urinary volume were not significantly changed in any of the groups studied (Table 1).

**Figure 1:**
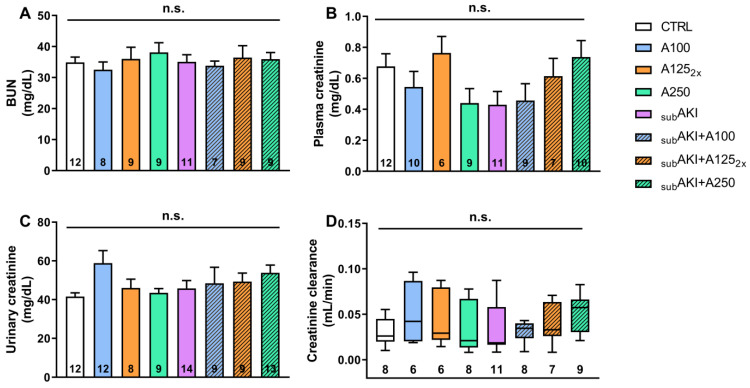
Effects of anethole treatment on glomerular renal function. Mice were randomly divided into eight experimental groups as detailed in the Materials and Methods (Section 4). (1) CTRL, mice treated with sterile saline solution via i.p. and water via gavage (v.g.); (2) A100, mice treated with anethole 100 mg/kg/day v.g., and saline via i.p.; (3) A125_2x_, mice treated with anethole 125 mg/kg, twice a day, v.g., and saline via i.p.; (4) A250, mice treated with anethole 250 mg/kg/day v.g. and saline via i.p.; (5) _SUB_AKI, mice treated with BSA via i.p. and water v.g.; (6) _SUB_AKI+A100, mice treated with both BSA and A100; (7) _SUB_AKI+A125_2x_, mice treated with both BSA and A125_2x_; (8) _SUB_AKI+A250, mice treated with both BSA and A250. (**A**) BUN (blood urea nitrogen) levels; (**B**) plasma creatinine concentration, (**C**) urinary creatinine concentration, and (**D**) creatinine clearance (CCr). The number inside the bar graph or boxplot represents the quantity of animals in each group (n). Columns represent the mean + standard error of the mean (SEM). Boxes show the interquartile (25–75%) range, and whiskers encompass a 10–90% range. n.s. represents not significance.

Glomerular function assessed by BUN (blood urea nitrogen), plasma and urinary creatinine, and creatinine clearance, biochemical parameters which are markers of the glomerular flow rate (GFR), were also not significantly changed (Figure 1A–D).

**Figure 2:**
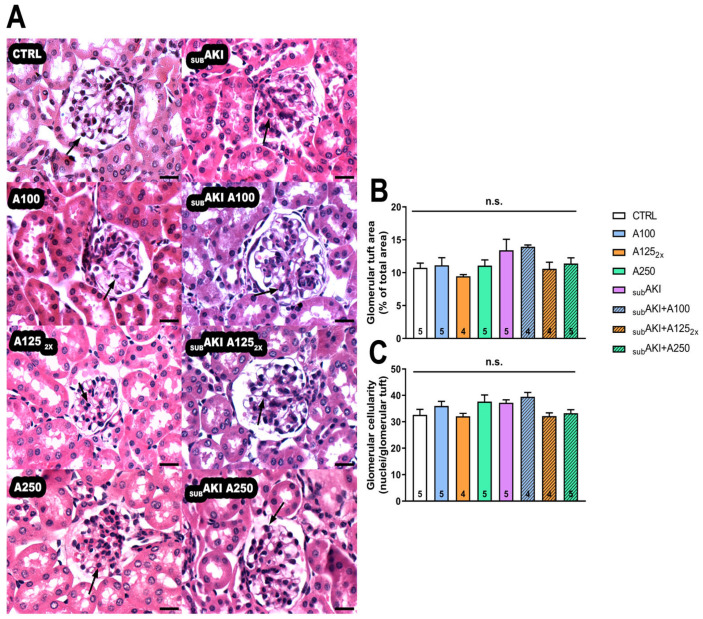
(**A**) Photomicrographs of glomerular area stained with hematoxylin–eosin. Mice were treated as described in Figure 1. (1) CTRL; (2) A100; (3) A125_2x_; (4) A250; (5) _SUB_AKI; (6) _SUB_AKI+A100; (7) _SUB_AKI+A125_2x_; (8) _SUB_AKI+A250. Thick arrows: cellular infiltrate in the glomerular tuft area. The number inside the bar graph represents the quantity of animals in each group (n). Bars = 100 µm and magnification = 40×. (**B**) Glomerular tuft area and (**C**) glomerular cellularity. Mice were treated as described in Figure 1. Columns represent the mean + standard error of the mean (SEM) and n.s. represents not significance.

Anethole also did not significantly change the histological parameters of glomerular tuft and cellularity (Figure 2A–C).

### 2.2. Effects of Anethole Treatment on Urinary Protein Excretion

**Figure 3:**
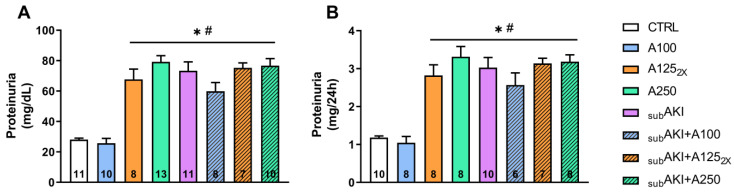
Effects of anethole treatment on urinary protein excretion. Mice were treated as described in Figure 1. (1) CTRL; (2) A100; (3) A125_2x_; (4) A250; (5) _SUB_AKI; (6) _SUB_AKI+A100; (7) _SUB_AKI+A125_2x_; (8) _SUB_AKI+A250. (**A**) Proteinuria (mg/dL). (**B**) Urinary proteins (mg/24 h). The number inside the bar graph represents the quantity of animals in each group (n). Columns represent the mean + standard error of the mean (SEM). * *p* < 0.05 versus CTRL. # *p* < 0.05 versus A100.

The proteinuria (mg/24 h) was significantly greater in the A125_2X_, A250, _SUB_AKI, _SUB_AKI+A100, _SUB_AKI+A125_2X_, and _SUB_AKI+A250 groups than in CTRL (1.182 ± 0.04; *p* < 0.001) and A100 (1.046 ± 0.16; *p* < 0.001). No significant difference was observed between CTRL and A100, which suggests that anethole, at the dose of 100 mg/kg, does not have a toxic renal effect (Figure 3A,B).

### 2.3. Effects of Anethole Treatment on Tubular Interstitial Cell Infiltrate

**Figure 4:**
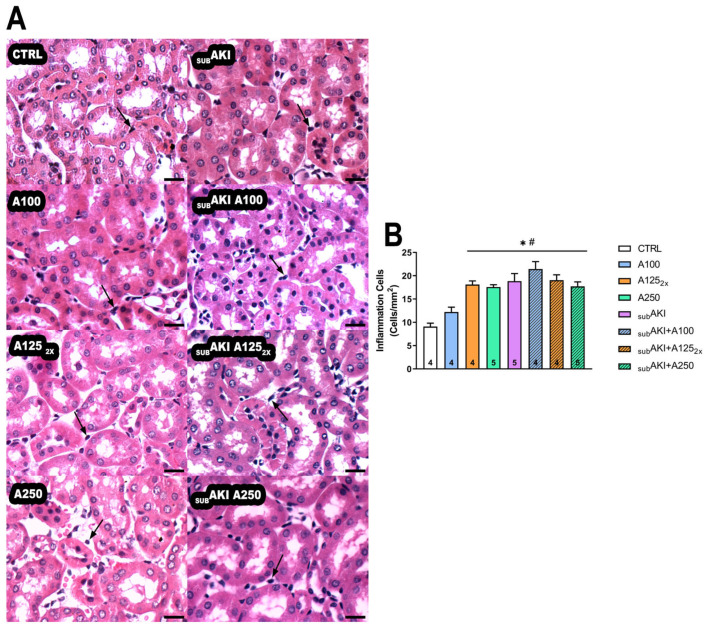
(**A**) Photomicrographs of renal interstitial cell infiltrate. Mice were treated as described in Figure 1. (1) CTRL; (2) A100; (3) A125_2x_; (4) A250; (5) _SUB_AKI; (6) _SUB_AKI+A100; (7) _SUB_AKI+A125_2x_; (8) _SUB_AKI+A250. Thick arrows: cellular infiltrate in the interstitial tubular space. The number inside the bar graph represents the quantity of animals in each group (n). Bars = 100 µm and magnification of 40×. (**B**) Inflammation cells. Mice were treated as described in Figure 1. Columns represent the mean + standard error of the mean (SEM). * *p* < 0.05 versus CTRL. # *p* < 0.05 versus A100.

The _SUB_AKI model is associated with tubular inflammation, and this was found here; the number of mononuclear cell counts was significantly higher in _SUB_AKI (*p* < 0.0001) compared to the CTRL group. This same increase in mononuclear cell counts was also observed in the anethole groups (A125_2X_ and A250) and all _SUB_AKI groups (_SUB_AKI+A100, _SUB_AKI+A125_2X,_ and _SUB_AKI+A250), as compared to CTRL (*p* < 0.001), but not in A100 (Figure 4A,B).

### 2.4. Effects of Anethole Treatment on in Vitro Albumin Endocytosis

**Figure 5:**
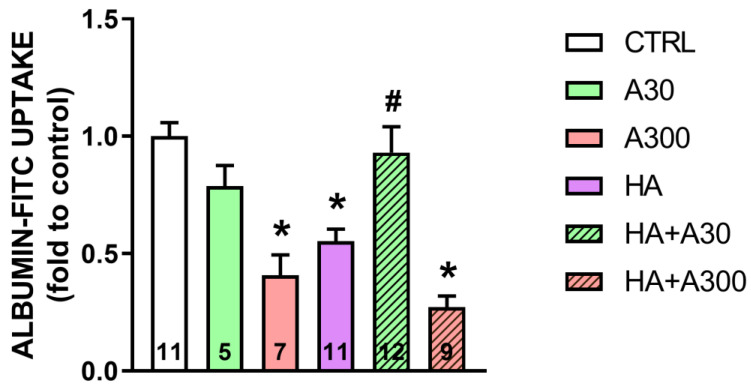
Effects of anethole treatment in vitro albumin endocytosis. CTRL group: albumin-FITC normal uptake in LLC-PK1 cells. A30 and A300 groups: cells treated with 30 µg/mL or 300 µg/mL of anethole. HA group: albumin-FITC uptake in LLC-PK1 cells with high albumin (100 mg/mL). HA+30 and HA+300 groups: cells with high albumin treated with 30 µg/mL or 300 µg/mL of anethole, respectively. The number inside the bar graph represents the quantity of cells in each group (n). Columns represent the mean + standard error of the mean (SEM). * *p* < 0.05 versus CTRL. # *p* < 0.05 versus A300, HA and HA+300.

A significant decrease in albumin endocytosis in LLC-PK1 cells, a model of PTECs, was observed in the A300 (*p* < 0.001), HA (*p* = 0.001) and HA+A300 (*p* < 0.001) groups (0.40 ± 0.08-, 0.55 ± 0.05-, and 0.27 ± 0.04-fold compared to the control, respectively) compared with the CTRL group (1.0 ± 0.05-fold compared to the control) (Figure 5). On the other hand, the HA+A30 group exhibited a significant increase in albumin endocytosis compared to the HA group, showing that anethole at 30 µg/mL was able to prevent changes in the endocytic machinery, since the values were 0.93 ± 0.11- and 0.55 ± 0.05-fold compared to the control, respectively (Figure 5).

### 2.5. Spontaneous Reversibility of Effects of Anethole Treatment on Tubulointerstitial Injury

**Figure 6:**
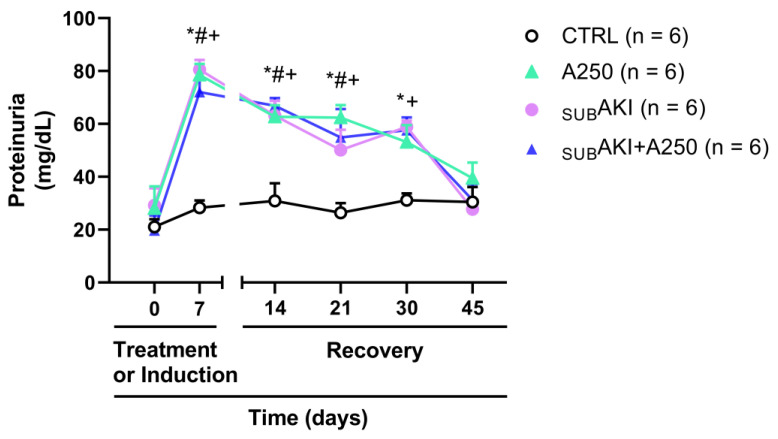
Reversibility of the effects of anethole treatment. Mice were treated as described in Figure 1. After this period, mice were housed in the animal facilities, and urine was collected on the 14th, 21st, 30th, and 45th days to assess proteinuria. (1) CTRL; (2) A250; (3) _SUB_AKI; (4) _SUB_AKI+A250. Data represent the mean + standard error of the mean (SEM). * *p* < 0.05 CTRL versus _SUB_AKI. # *p* < 0.05 CTRL versus A250. + *p* < 0.05 CTRL versus _SUB_AKI+A250.

On the 7th day, proteinuria was significantly higher in the A250, _SUB_AKI, and _SUB_AKI+A250 groups (78.50 ± 4.24; 80.54 ± 3.63; and 72.03 ± 5.12 mg/dL; *p* < 0.001) than in the CTRL group (30.45 ± 5.71 mg/dL), but did not differ among them (Figure 6). To assess whether the anethole effect was reversible, after completing the 7 days of treatment with anethole 250 mg/kg, proteinuria was assessed for 4 additional weeks (Figure 6). Indeed, at the 45th day, the proteinuria levels in the A250, _SUB_AKI and _SUB_AKI+A250 groups (39.43 ± 5.91; 27.69 ± 2.05; and 31.21 ± 5.99 mg/dL, respectively) returned to values similar to the CTRL group (30.45 ± 5.71 mg/dL), demonstrating that the toxic effect of anethole at a dose of 250 mg/kg was temporary.

## 3. Discussion

### 3.1. Major Discoveries

The major discovery of this investigation is that in vivo acute treatment with a high dose of anethole (250 mg/kg) promotes tubulointerstitial renal injury, with proteinuria and mild toxicity which is spontaneously reversible in four weeks. Moreover, in vitro experiments demonstrated that a high concentration of anethole (300 µg/mL, approx. 2 mM) inhibits the uptake of albumin by tubular cells in culture, thus suggesting a mechanism for proteinuria causation. However, at smaller doses in in vivo experiments (100 mg/kg), anethole induced no toxicity. Administered in vitro at smaller concentrations (30 µg/mL approx. 0.2 mM), anethole showed an effect opposite to that observed at a high dose: it antagonized the inhibition, induced by the high albumin concentration in the culture medium, of albumin uptake by tubular cells. To our knowledge, the data presented herein are novel.

### 3.2. Renal Effect of High Doses of Anethole: Lesion Restricted to Tubules and Tubular Inflammation

The proteinuria induced by 250 mg/kg of anethole (approximately 3 mg/24 h) (Figure 3B) represented approximately a three-times increase compared to the control level (approximately 1 mg/24 h) and appeared quantitatively relevant since it reached a magnitude similar to that observed with the _SUB_AKI model. Interestingly, like the proteinuria of the _SUB_AKI model, that induced by anethole was fully spontaneously reversible in four weeks (Figure 6) and, in turn, it was not additive with that of the _SUB_AKI model (Figure 3A,B). Several studies have shown that proteinuria can trigger inflammation [35,36,37], and the proteinuria here observed could be partly due to high peak blood concentrations following a single 250 mg/kg daily dose of anethole; the fractionation of this dose was investigated. Thus, the dose was fractionated into two daily administrations of 125 mg/kg each, 10 h apart, and this did not change the proteinuria level. These findings are consistent with the literature, as experimental studies in rats indicate that anethole is entirely but slowly absorbed after oral administration [2].

Moreover, the absence of additional nephrotoxicity with the 250 mg/kg dose in the _SUB_AKI model (Figure 3 and Figure 4, _SUB_AKI+A250 compared to the _SUB_AKI group) suggests a ceiling effect in anethole-induced renal toxicity or a possible adaptive renal response. Another reasonable hypothesis is that at 250 mg/kg, compensatory mechanisms such as enhanced renal clearance or metabolic adaptation may mitigate further injury, preventing additional nephrotoxicity in the presence of albumin-induced stress.

As expected, acute treatment with anethole and the _SUB_AKI model did not alter renal parameters, body mass, water, or food intake (Table 1), nor did it change the glomerular function (Figure 1). Similar results were also found in studies with anethole and EOCz carried out by our group [26,27]. Because proteinuria was more related to tubular function, anethole and/or high albumin were investigated for their potential to generate histological impairments. The highest (250 mg/kg) and the fractionated (125 mg/kg, daily twice) dose of anethole increase renal tubular cell infiltration, and the same effect was observed in the _SUB_AKI group induced by albumin administration (Figure 4A,B). 

### 3.3. Possible Explanation of Mechanistic of Action

Concerning the mechanism of action, it is important to notice that no signs of histological glomerular alteration were detected. Therefore, the present study can suggest two hypotheses to explain the proteinuria: either anethole had a primary pro-inflammatory effect at the level of the renal tubules, which caused inflammation, and subsequently proteinuria, or it was primarily inhibiting the tubular uptake and transport of protein from the lumen to the interstitial space, and secondarily causing inflammation [38]. Since anethole is considered a powerful anti-inflammatory agent, the hypothesis of a primary inflammatory effect restricted to the tubules was considered less probable [2].

Further considering the mechanism of action, the present study shows that high concentrations of anethole (300 µg/mL in vitro, 250 mg/kg in vivo) inhibit albumin uptake in tubular cells. This inhibition may occur because anethole interferes with megalin’s function, either by directly binding to the receptor or by disrupting its signaling pathways. As a result, albumin is not effectively reabsorbed and is instead excreted in the urine, leading to proteinuria. Over time, the excessive protein in the tubular lumen may trigger tubular interstitial inflammation, which is observed in histological analyses. However, since the damage is limited to the tubules and does not involve glomerular alterations, suggesting the primary effect is at the level of tubular reabsorption, the kidney appears capable of recovering spontaneously once anethole is cleared from the system.

Another hypothesis to be considered is that high doses of anethole may induce metabolic stress or oxidative damage in proximal tubular cells, since terpenes and terpenoids at high concentrations may cause oxidative stress [39]. Anethole has known antioxidant properties at lower doses, but paradoxically, at higher doses, it might overwhelm cellular detoxification pathways, eventually leading to increased reactive oxygen species production [40]. The oxidative stress may transiently impair endocytic vesicle trafficking, further reducing albumin uptake [41,42]. Once anethole is metabolized and cleared, antioxidant defenses recover, restoring normal tubular function, and explaining the spontaneous reversibility of proteinuria seen in the present study.

### 3.4. The _SUB_AKI Model

The similarity of the proteinuria induced by anethole and that of the _SUB_AKI model deserves consideration. Concerning the _SUB_AKI model, this is characterized by an acute tubule-interstitial injury without changes in glomerular function and structure [27,43,44,45]. Remarkably, it is known that protein reabsorption occurs through an endocytosis mechanism by the PTECs, among these mechanisms, receptor-mediated endocytosis is the main mechanism associated with this process [46,47,48]. The mechanism of receptor-mediated endocytosis involves the activation of a complex of receptors composed of megalin, cubilin, and amnionless, but only megalin has a cytoplasmic portion, being proposed to regulate the endocytic process [49]. Changes in the endocytic machinery, which involves megalin, are documented to be able to cause proteinuria [50]. The similarity of parameter magnitude in the anethole effect and the _SUB_AKI model suggests that both share common biochemical steps in the mechanism of action.

AKI in humans affects one in five hospitalized patients worldwide and is associated with high mortality, proteinuria being the pathognomonic parameter and observable at the initial stages [28,29,51,52]. The similarity among these cases of proteinuria suggests a shared pathway in the cascade of the mechanism of action.

### 3.5. Effects of Anethole on Tubular Albumin Uptake in Vitro and Renal Effect of Low Doses of Anethole

To distinguish between a primary versus a secondary interference in the tubular uptake of luminal protein, the in vitro albumin uptake by tubular cells in culture was measured. Remarkably, the results demonstrated a primary interference with protein uptake. Anethole, at 300 µg/mL, a concentration considered high as related to the promotion of several effects, inhibited albumin uptake. This inhibition was quantitatively similar to that induced by high albumin concentration (100 µg/mL, HA group). The combined presence of anethole (300 µg/mL) and a high albumin concentration (100 µg/mL) did not show a clear additive effect (Figure 5, comparison of groups A300 with HA+300). Thus, concerning the comparisons between the effects of anethole and a high concentration of protein on tubular uptake, the effects in vitro showed several similarities to those in vivo. This reinforces the suggestion that, in vivo, anethole has a primary inhibitory effect on the tubular uptake of luminal proteins.

A rich portfolio of pharmacological activities with interesting potential therapeutic implications has been described for anethole and EOCz [4,5,18,20,26]. Amongst these activities, the neuroprotective activity of high doses of EOCz (300 mg/kg) in diabetic neuropathy has shown a very promising perspective (patent BR 1020180758-1) [20,21]. Because of this, an investigation of the pharmacological effects of relatively high doses of anethole (which comprises 80% of EOCz) was undertaken. It was also undertaken to verify whether the renal effects of EOCz were primarily determined by its anethole content or whether these effects received important modificatory contributions of other constituents. Considering the data here presented and those previously published with EOCz, it is reasonable to infer that the renal effects of high doses of EOCz are predominantly determined by the anethole content of this essential oil.

Smaller doses of EOCz (100 mg/kg) also showed promising protective effects against diabetic neuropathy (patent BR 1020180758-1). Thus, it was interesting to discover that dosed at 100 mg/kg, anethole induced no proteinuria (Figure 3, A100 group) and no inflammation (Figure 4, A100 group). Since anethole is an effective anti-inflammatory agent in the range of doses 1–30 mg/kg [5], this suggests that anethole could have anti-inflammatory activity without any renal toxicity. On the other hand, 100 mg/kg of anethole did not offer a protective effect against the _SUB_AKI model (Figure 3 and Figure 4, _SUB_AKI+A100 group). The _SUB_AKI model primarily induces tubular overload due to increased plasma protein levels, which could overwhelm any potential renoprotective effects of anethole at 100 mg/kg.

Additionally, nephroprotection often require mechanisms beyond anti-inflammatory effects, such as the preservation of glomerular filtration barrier integrity. Along these lines, 100 mg/kg may not have been sufficient to exert these additional protective actions. Future studies with lower doses (<100 mg/kg) and mechanistic investigations could help to determine whether anethole has a protective window or if its effects are dose-dependent beyond a certain threshold.

Surprisingly, in vitro, the experiments with albumin uptake by tubular cells demonstrated that a relatively small concentration of anethole (30 µg/mL, approximately 200 µM) does not affect albumin uptake by tubular cells (Figure 5, comparison of groups A30 with CTRL). Indeed, it also promoted an effect opposite to that of high concentration, i.e., was able to prevent the decrease in extracellular protein uptake (Figure 5, comparison of HA+A30 with HA or HA+A300). Since anethole is an effective anti-inflammatory agent in the range of doses 1–30 mg/kg, this suggests that anethole could have anti-inflammatory activity without any renal toxicity.

### 3.6. Therapeutic Potential of Anethole: Animal Models and Human Outcomes

The treatment of diabetic animals with anethole (300 mg/kg, 4 weeks) prevented morphological, electrophysiological, and oxidative stress abnormalities induced by diabetes in the sciatic nerve of rats [53]. Lima et al. [18], demonstrated that EOCz, anethole, and estragole act as important antispasmodic agents on tracheal muscle. The administration of trans-anethole (10 and 50 mg/kg/day, i.p., 10 days) has a potent inhibitory activity on Escherichia coli LPS-induced periodontitis in rats, exerting an anti-inflammatory effect that is similar to ketoprofen, a non-steroidal anti-inflammatory drug (10 mg/kg, i.p.) [54]. The EOCz (300 mg/kg, orally) was able to reverse the lesion in the respiratory system of mice submitted to the OVA-induced asthma model and its antioxidant action was likely the main mechanism of action in the reversal of this lesion [10]. Furthermore, a previous sub-acute study carried out by our group showed that anethol and EOCz, administered *per os* for longer than ten weeks, triggered toxicity at 250 mg/kg [26].

To the best of our knowledge, no clinical trials have been conducted on anethole; however, in vitro studies suggest its potential as an anticancer therapy. In humans, anethole shows potential as an antitumor agent by interfering with cancer cells and exhibiting pro-apoptotic, anti-metastatic, and anti-inflammatory effects [55]. It induces apoptosis in human breast cancer cells [56], as well as in cervical carcinoma [57]. Additionally, anethole inhibits cell migration and invasion in human fibrosarcoma [12] and prostate cancer cells [58].

Finally, based on the aforementioned data, it is clear that anethole exhibits significant anti-inflammatory and antioxidant properties, highlighting its potential as a therapeutic agent. However, long-term clinical studies are necessary to confirm its effectiveness, delineate the safety profile, and develop strategies to mitigate any adverse effects.

## 4. Materials and Methods

### 4.1. Animals

One hundred and fifteen male Swiss mice (8 weeks old), from the Vivarium of the Institute of Biomedical Sciences—Universidade Estadual do Ceará (UECE), were kept at a constant temperature (22 ± 2 °C) in a 12 h/12 h light/dark cycle with free access to standard chow and water. All animal procedures were conducted following the National Institutes of Health Guide for the Care and Use of Laboratory Animals and to ARRIVE guidelines and the National Council for the Control of Animal Experimentation, Ministry of Science, Technology, and Innovation (CONCEA/MCTI), Brazil. All experimental protocols were reviewed and approved by the Institutional Ethics Committee of UECE (protocol number 00128900/2021, 20 May 2021).

### 4.2. Subclinical AKI Animal Model and Anethole Treatment

**Figure 7:**
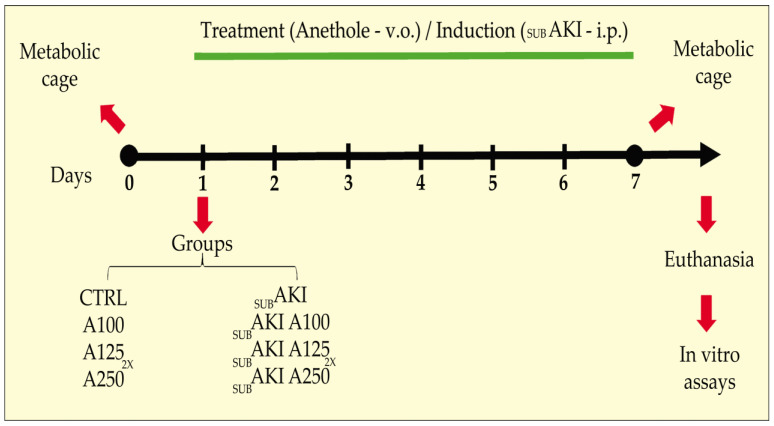
Schematic diagram of the study design. Mice were randomly divided into eight experimental groups as detailed in the Materials and Methods (Section 4). CTRL: control, mice treated daily with vehicles). A100, A125_2x_, A250: mice treated daily with 100, 125 twice daily, and 250 mg/kg anethole, respectively. _SUB_AKI: subclinical acute kidney injury mice. _SUB_AKI+A100, _SUB_AKI+A125_2x_, _SUB_AKI+A250: subclinical acute kidney injury mice and treated daily with anethole 100, 125 twice a day, and 250 mg/kg, respectively. V.g.: via gavage (*per os*). i.p.: intraperitoneal.

The development of the subAKI animal model and anethole (99% purity, Sigma-Aldrich) treatment was performed as previously published [27,44]. Briefly, mice were divided randomly into 8 different groups: (1) Control (CTRL), mice treated with sterile saline solution (0.9% NaCl, 37 °C), used as a vehicle for bovine serum albumin (BSA), via i.p., and water (used as a vehicle for anethole), via gavage; (2) A100, mice treated with anethole 100 mg/kg/day via gavage, and saline via i.p.; (3) A125_2x_, mice treated with anethole 125 mg/kg twice daily, via gavage, and saline via i.p.; (4) A250, mice treated with anethole 250 mg/kg/day via gavage, and saline via i.p.; (5) _SUB_AKI, mice treated with BSA 10 g/kg/day via i.p. and water via gavage; (6) _SUB_AKI+A100, mice treated with both BSA and A100; (7) _SUB_AKI+A125_2x_, mice treated with both BSA and A125_2x_; (8) _SUB_AKI+A250, mice treated with both BSA and A250. All groups were treated daily for 7 consecutive days (Figure 7). The dose of anethole used was based on an expected good compromise between appropriate pharmacological efficacy for neuropathic protective effects and toxicity, as shown by previous studies conducted by our group [26,27,53].

On day 7 of treatment, the mice were housed in metabolic cages for 24 h. Body mass, water, food, and urine output were measured. The mice were then euthanized with a mixture of ketamine (100 mg/kg) and xylazine (10 mg/kg). Blood samples were obtained by cardiac puncture. The left kidneys were perfused with heparinized saline and 4% paraformaldehyde using a peristaltic pump and extracted for further analysis.

An additional batch of twenty mice was used to analyze the reversibility of the effects of anethole treatment. The same 7-day induction and treatment protocol previously described was applied in this case. After this period, the mice were housed in the animal facilities, and urine was collected on the 14th, 21st, 30th, and 45th days following the start of the protocol to assess proteinuria.

### 4.3. Renal Function Analysis

Renal function was analyzed as described previously [27]. The measurements described below were performed in sample plasma and urine, using commercial kits available. In brief, 24 h urine samples were quantified to determine urinary flow (mL/min), proteinuria, and urinary creatinine. Then, the samples were clarified by 5 cycles of centrifugation (10,000× *g* for 10 min) to remove urine sediments. Immediately after withdrawal, the blood was placed in a heparinized tube and centrifuged (3500× *g* for 10 min). The plasma and urinary creatinine concentrations were measured using the alkaline picrate method (Labtest kit no. 35; Belo Horizonte, MG, Brazil). Blood urea nitrogen (BUN) was determined by the urease method (Labtest kit no. 27; Belo Horizonte, MG, Brazil). The urinary protein concentration was determined by the Pyrogallol Red method (Labtest kit no. 36; Belo Horizonte, MG, Brazil). All analyses were made following the manufacturer’s instructions. The creatinine clearance (CCr) parameter was calculated as described previously by our group [59].

### 4.4. Histological Analyses

Histological analyses were performed as previously published [27,59,60]. Briefly, the kidneys were fixed in a 4% buffered formalin solution and embedded in paraffin, and histologic sections (5-μm-thick) were obtained and stained with hematoxylin–eosin (Sigma-Aldrich, St Louis, MA, USA), to evaluate the glomerular tuft area and glomerular and interstitial cellularity.

To obtain the quantitative data images of the kidney, sections were acquired using a Nikon E800 Eclipse light microscope (Nikon, Japan) coupled to a digital camera Evolution VF (Media Cybernetics, Rockville, MD, USA). The interface capture software used was a Q-Capture 2.95.0, version 2.0.5 (Silicon Graphics Inc, Milpitas, CA, USA); from each animal, 15 high-resolution randomly taken images (2048 × 1536 pixel buffer) were transmitted to a color LCD monitor in TIFF format and digitized. Images were analyzed with Image-Pro Plus software version 7.0.1.658 (Media Cybernetics, Rockville, MD, USA).

The glomerular tuft area was determined by directly measuring the glomerular tuft area, expressed as a percentage of the total area. Inflammation was calculated as the percentage of inflammatory cells present in the interstitium or glomeruli concerning the total tissue or glomerular area.

### 4.5. Cell Culture

LLC-PK1 cells (ATCC, Manassas, VA, USA), a porcine cell line of proximal tubule epithelial cells, were maintained in a cell culture incubator with 5% CO_2_ and cultured in low-glucose DMEM (Life Technologies, Carlsbad, CA, USA). Culture media were supplemented with 10% previously heat-inactivated fetal bovine serum (FBS) and 1% antibiotic–antimycotic. After 2 days of cultivation, cells were starved (low-glucose DMEM in the absence of FBS) and incubated overnight with 30 or 300 µg/mL of anethole (Sigma-Aldrich, Saint Louis, MO, USA) to follow the experimental assay and evaluate albumin-FITC endocytosis.

### 4.6. Albumin-FITC Endocytosis Assay

Albumin-FITC endocytosis assays were carried out as previously described [61,62,63]. LLC-PK1 cells were washed 3 times with Ringer’s solution (20 mM HEPES-Tris, pH 7.4, 140 mM NaCl, 2.7 mM KCl, 1.8 mM CaCl_2_, 1 mM MgCl_2_, 5 mM d(+)-glucose). After the wash process, cells were incubated with a solution containing 100 μg/mL albumin-FITC (delipidated, #A9647, Sigma-Aldrich, St. Louis, MO, USA) diluted in Ringer’s solution at 37 °C for 30 min. To assess specific receptor-mediated endocytosis, cells were co-incubated with 100 mg/mL of non-labeled BSA to determine receptor-independent endocytosis, and the referring values were discounted. After 30 min, LLC-PK1 cells were kept on ice and washed 10 times with Ringer’s solution to remove unbound albumin-FITC. Cells were lysed using ice-cold lysis buffer containing 20 mM MOPS (pH 7.4) and 0.1% Triton X-100. The homogenate of these cells was collected, and cell-associated fluorescence intensity was analyzed using a microplate spectrofluorometer (SpectraMax M5; Molecular Devices, Sunnyvale, CA, USA). Importantly, the cell-associated fluorescence was normalized to the total protein concentration of each sample, and evaluated using the Folin phenol method. Data are expressed as fold change compared to controls.

### 4.7. Statistical Analysis

Graphics and statistical analyses were performed using GraphPad Prism 8 (version 8; GraphPad Software, San Diego, CA, USA). Statistical differences among the experimental groups were determined by a one-way analysis of variance test followed by Tukey’s multiple comparisons test or Kruskal–Wallis for non-parametric tests. *p* < 0.05 was used to determine statistical significance. Parametric data were expressed as the mean and standard error of the mean (SEM), while non-parametric data were represented by the median (interquartile range).

## 5. Conclusions

In conclusion, the present study demonstrated that anethole at high doses bears a renal acute toxicity that, although mild and spontaneously fully reversible, ought to undergo a cost–benefit analysis. On the other hand, it also demonstrates that for the obtention of some important effects, like anti-inflammatory effects, which reach full efficacy at doses below 100 mg/kg, anethole may be employed without renal toxicity. Since this study was performed in mice, it is not warranted to assume that these effects hold for other animal species, and they need to be confirmed in humans. The similarity between the results of anethole and EOCz suggests that this essential oil has the same characteristic of reversibility and upper bound dose for the absence of renal toxicity, but this remains to be experimentally determined. Finally, long-term clinical studies are needed to validate the usefulness of anethole.

This study presents limitations: (1) There are no conditions to further elucidate the mechanism of anethole-induced proteinuria with the data gathered. (2) The redox status was not measured. (3) Doses lower than 100 mg/kg were not tested. (4) The study was carried out on an animal model and there is no guarantee that it will apply to humans.

## 6. Patents

BR 1020180758-1.

## Figures and Tables

**Table 1:** Effect of anethole on renal parameters, body mass, water, and food intake.

Groups	Body Mass (g)	Absolute Kidney Weight (g)	Relative Kidney Weight (%)	Food Intake (g)	Water Intake (mL)	Urinary Volume (µL)
CTRL	30.50 ± 0.77	0.322 ± 0.01	1.059 ± 0.03	4.863 ± 0.28	7.21 ± 0.69	700.0 ± 89.61
A100	29.73 ± 0.83	0.310 ± 0.01	1.034 ± 0.05	4.512 ± 0.32	6.60 ± 0.47	787.5 ± 47.95
A125_2x_	30.77 ± 0.61	0.326 ± 0.01	1.062 ± 0.04	4.382 ± 0.53	6.87 ± 0.89	766.7 ± 126.9
A250	31.56 ± 0.81	0.367 ± 0.01	1.169 ± 0.06	4.542 ± 0.23	7.83 ± 0.51	677.8 ± 99.69
_SUB_AKI	28.37 ± 0.57	0.312 ± 0.01	1.103 ± 0.05	4.567 ± 0.51	6.32 ± 0.26	636.4 ± 85.57
_SUB_AKI+A100	30.03 ± 0.94	0.334 ± 0.01	1.126 ± 0.04	4.620 ± 0.33	7.21 ± 0.53	875.0 ± 133.3
_SUB_AKI+A125_2x_	28.89 ± 1.08	0.336 ± 0.01	1.170 ± 0.03	4.572 ± 0.17	7.07 ± 0.80	766.7 ± 88.19
_SUB_AKI+A250	30.12 ± 1.29	0.366 ± 0.01	1.223 ± 0.04	3.891 ± 0.27	5.90 ± 0.64	700.0 ± 91.29

Mice were randomly divided into eight experimental groups as detailed in the Materials and Methods (Section 4). CTRL: control, mice treated daily with vehicles (saline solution and water). A100, A125_2x_, A250: mice treated daily with 100, 125 twice a day and 250 mg/kg anethole, respectively. SUBAKI: subclinical acute kidney injury mice. _SUB_AKI+A100, _SUB_AKI+A125_2x_, _SUB_AKI+A250: subclinical acute kidney injury mice and treated daily with anethole 100, 125 twice a day, and 250 mg/kg, respectively. Data are represented as the mean + standard error of the mean (SEM) of 8–13 animals in each group.

## Data Availability

The data that support the findings of this study are available from the corresponding author upon reasonable request.
